# Valve-in-valve as a rescue treatment in retrograde migration of the transcatheter aortic valve to the left ventricle: a case report

**DOI:** 10.1093/ehjcr/ytad554

**Published:** 2023-11-07

**Authors:** Juan F Garcia-Garcia, Jose R Gayosso-Ortiz, Roberto Muratalla-Gonzalez, Juan C Fuentes-Moreno, Heberto Aquino-Bruno

**Affiliations:** Interventional Cardiology Service, General Hospital of Mexico, Dr. Balmis 148, Col. Doctores, Cuauhtémoc, 06720 Mexico City, Mexico; Interventional Cardiology Service, National Medical Center "November 20", Av. Felix Cuevas #540, Col. Del Valle Del. Benito Juarez, Mexico City 03100, Mexico; Interventional Cardiology Service, General Hospital of Mexico, Dr. Balmis 148, Col. Doctores, Cuauhtémoc, 06720 Mexico City, Mexico; Interventional Cardiology Service, National Medical Center "November 20", Av. Felix Cuevas #540, Col. Del Valle Del. Benito Juarez, Mexico City 03100, Mexico; Interventional Cardiology Service, National Medical Center "November 20", Av. Felix Cuevas #540, Col. Del Valle Del. Benito Juarez, Mexico City 03100, Mexico; Interventional Cardiology Service, National Medical Center "November 20", Av. Felix Cuevas #540, Col. Del Valle Del. Benito Juarez, Mexico City 03100, Mexico

**Keywords:** Case report, Aortic valve regurgitation, TAVR, Valve migration, Valve-in-valve

## Abstract

**Background:**

The treatment of choice for patients with severe symptomatic pure native aortic valve regurgitation (PNAVR) is surgical aortic valve replacement (SAVR). However, not all patients are candidates for surgery because of comorbidities or are deemed high risk for surgery. In such cases, transcatheter aortic valve replacement (TAVR) has proved to be better than medical treatment.

**Case summary:**

A 78-year-old male with a history of ankylosing spondylitis was admitted with New York Heart Association III heart failure. The echocardiogram showed severe aortic regurgitation and a left ventricular ejection fraction of 52%. Because of high surgical risk and being refractory to medical RX, he was accepted for TAVR. The tomography of anatomical characteristics reported the absence of calcium and dilation of the aortic ring and aortic root. During the TAVR procedure, the patient experienced valve migration, but it was autonomously repositioned in the aortic annulus. As a rescue measure, a second valve was placed. Here, we present a case of valve migration to the left ventricle treated with a valve-in-valve procedure without the need for surgical treatment.

**Discussion:**

The absence of annulus calcification in PNAVR increases the risk of post-TAVR paravalvular leak and device embolization. Valve migration generally requires valve recovery and conversion to SAVR.

**Learning points**

- In patients with pure native aortic valve regurgitation and giant rings, balloon-expandible transcatheter aortic valve replacement is a feasible option, with oversizing >15% in order to avoid the risk of valve migration.
- Valve migration with autonomous inverse reposition carries the risk of obstructive shock and cardiac arrest. In this situation, immediate introduction of the guidewire into the left ventricle should be considered with the possibility of performing a valve-in-valve procedure.

## Introduction

Pure native aortic valve regurgitation (PNAVR) is characterized by severe aortic valve regurgitation without an element of aortic stenosis, a valve area >1.5 cm^2^, and no calcification on leaflets or annulus.^1^

The treatment of choice for patients with severe symptomatic PNAVR is surgical aortic valve replacement (SAVR).^2^ However, not all patients are suitable candidates for surgery due to comorbidities or are deemed high risk. In this case, multiple studies have shown that transcatheter aortic valve replacement (TAVR) has better outcomes than medical treatment alone for patients with inoperable PNAVR.^3,4^

In severe PNAVR cases, the absence of annulus calcification poses a greater challenge in anchoring and stabilizing the device during deployment, consequently increasing the risk of post-TAVR paravalvular leak and device embolization.^5^

Retrograde valve migration (RVM) of the prosthetic valve after TAVR is very rare. A concomitant presentation of annular and aortic root dilatation, combined with the suction effect by the left ventricle (LV), contributes to RVM.^5^

## Summary figure

**Table:** 

Date	Events
September 2022	A 78-year-old male with a history of heart failure and functional class New York Heart Association III was recently diagnosed with pure native aortic valve regurgitation
October 2022	At a meeting of the heart team, it was decided that, due to high surgical risk, he should be managed with medical treatment
February 2023	Unfortunately, there was no improvement in his clinical state despite medical treatment
February 2023 Day 1	At a new meeting held by the heart team, it was decided to proceed with percutaneous treatment through transcatheter aortic valve replacement
February 2023 Day 2	Because of anatomical characteristics, an Edwards #29 expandable balloon valve was placed
February 2023 Day 2	After percutaneous aortic valve placement, he presented with migration to the left ventricle, with autonomous repositioning in the aortic annulus
February 2023 Day 2	Cardiopulmonary resuscitation was performed for 6 min, at which time a second valve was placed in the aortic position as a rescue measure
February 2023 Day 2	Return of spontaneous circulation was achieved with the improvement of anterograde flow
February 2023 Day 2	Control aortography revealed a return of anterograde flow and adequate filling
Day 2	Control echocardiogram showed a stable valve with no leak or aortic regurgitation
Day 5	The patient was discharged on Day 5 after the procedure, without any neurological damage

## Clinical case

A 78-year-old male with a history of Type 2 diabetes and hypertension was being treated with a non-dihydropyridine calcium antagonist and thiazides. He was admitted to our centre with decompensated heart failure Class III New York Heart Association (NYHA). During the physical examination, an early diastolic decrescendo murmur was heard best at the third intercostal space on the right. The echocardiogram revealed severe aortic regurgitation, with a central regurgitant jet, dense vena contracta measuring 7 mm, pressure half-time of 188 ms, effective regurgitant orifice area of 32, and regurgitant volume of 62 mL. There was also dilatation of the aortic root and a left ventricular ejection fraction (LVEF) of 52% with a left ventricular end-systolic diameter of 26 mm/m^2^.

During the use of the aetiological approach, bicuspid pathology and infective endocarditis were ruled out. The coronary angiography showed no angiographic lesions. However, due to the patient’s high surgical risk (STS score 8.1% and frailty), the heart team decided to continue medical treatment by adding angiotensin-converting enzyme inhibitors (ACEIs). Unfortunately, there was no improvement in the patient’s clinical state. Therefore, TAVR was considered the best approach in this scenario.

**Figure 1:**
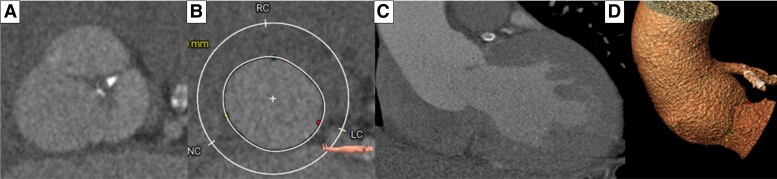
Cardiac computed tomography. (*A*) Tricuspid aortic valve with an absence of calcium in the leaflets. (*B*) A giant ring with an absence of calcium. (*C* and *D*) A dilation of the aortic root and ascending aorta can be observed.

During the planning of the procedure, a tomography revealed dilatation of the aortic root measuring 50 mm in the sinuses (non-coronary sinus 43 mm, left coronary sinus 41 mm, right coronary sinus 42 mm) and an annulus perimeter of 105 mm (*Figure 1*). There was no presence of calcium (*Figure 1*). These characteristics indicated a high risk of migration, so a decision was made to use the Edwards SAPIEN 3 valve #29.

**Figure 2:**
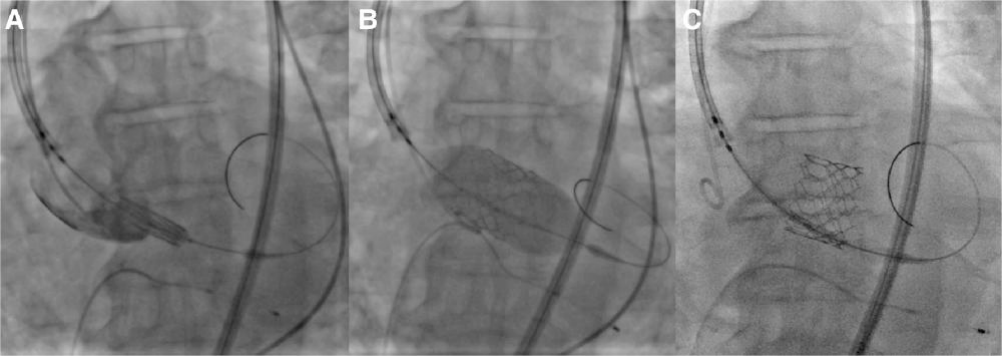
(*A*) Placement of a pigtail catheter in the non-coronary sinus. (*B*) Valve deployment with 15% oversizing. (*C*) Immediate migration to the left ventricle.

**Figure 3:**
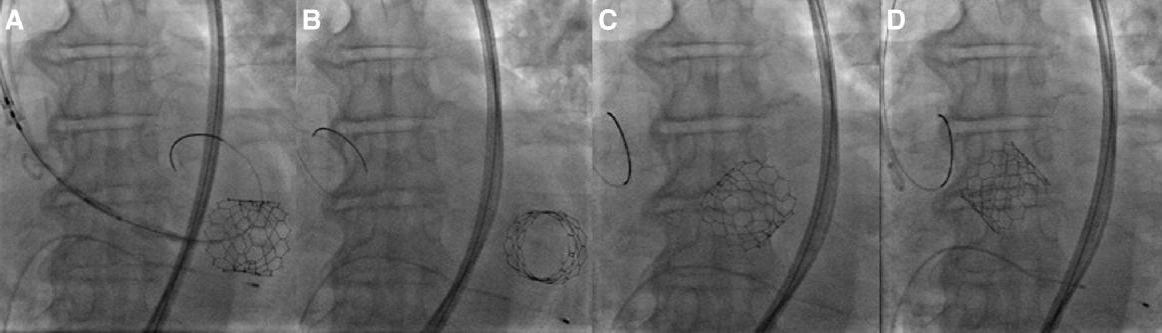
(*A*–*C*) Valve migration to the left ventricle, with a 180° rotation inside the ventricular cavity. (*D*) Autonomous insertion in the aortic annulus in reverse valve position.

**Figure 4:**
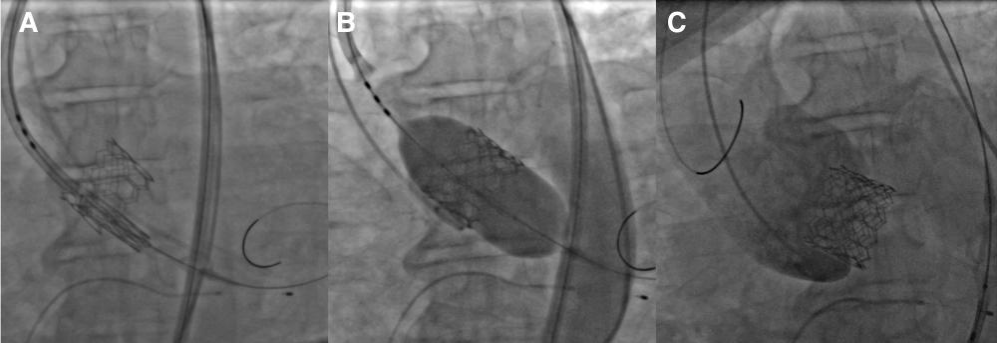
Step-by-step procedure. (*A*) Alignment of the second valve. (*B*) Aortic valve-in-valve without complications. (*C*) Adequate antegrade flow with no leakage or valve regurgitation.

With a bilateral femoral arterial approach and under sedation, with pacing at 180 b.p.m., the Edwards SAPIEN 3 valve #29 was placed, with an oversizing of 15% (*Figure 2A* and *B*). Unfortunately, during the post-deployment control aortography, the aortic valve migrated retrogradely into the LV (*Figure 2C*; Supplementary material online, *Video S1*). After removal of the Lunderquist guidewire, rotational movement of the valve was observed in the ventricular cavity, autonomously introducing itself into the aortic annulus of the LV in reverse position (*Figure 3A–D*; Supplementary material online, *Video S2*). After this unexpected complication, the patient immediately became hypotensive, and this was arrested with pulseless electrical activity. We started cardiopulmonary resuscitation (basic life support) for 6 min, and the Lunderquist guidewire was promptly introduced to displace the leaflets and improve antegrade flow. To address the situation, we decided to implement a rescue measure by placing a second valve. Through the Lunderquist guidewire, the second valve was deployed with annular implantation and an oversizing of 20% (*Figure 4A–C*; Supplementary material online, *Video S3*). Control aortography showed adequate placement with the normal antegrade flow without the presence of leakage or valve insufficiency (see Supplementary material online, *Video S4*). Post-procedure, echocardiographic parameters reported a mean gradient of 8 mmHg and a velocity of 1.8 m/s. The patient was discharged on Day 5 after the procedure, without any neurological damage.

## Discussion

Current guidelines suggest that patients with severe aortic regurgitation who are symptomatic or asymptomatic with an LVEF <50% should undergo SAVR. Conversely, patients who are unable to undergo SAVR due to contraindications are to be treated conservatively with medical therapy.^2^ Medical therapy, especially the use of ACEIs or dihydropyridines, may provide symptomatic improvement for individuals with chronic severe aortic regurgitation, for whom surgery is not a viable option.^2^ However, the results of the efficacy of medical therapy in the treatment of PNAVR are unsatisfactory.^6^ Patients with severe PNAVR (NYHA Class III or IV) who are on medical treatment face an alarming mortality rate of nearly 25% a year.^6^ These findings highlight the fact that there is an unmet clinical need for patients with inoperable PNAVR.

Various studies have shown that TAVR has better outcomes than medical treatment for patients with inoperable PNAVR.^3,4^ However, TAVR is used in only a fraction of patients with aortic regurgitation. This is in part due to the lack of aortic valve calcification in many patients with aortic insufficiency, making anchoring of the new valve cumbersome. As a result, TAVR is being used as an off-label procedure in patients with PNAVR.^3^

RVM is a rare, yet a potentially life-threatening complication that can occur either during or after TAVR.^7^ Among the risk factors, we can identify the following: the absence of aortic annular calcification, failure to perform computed tomography TAVR measurement, low transcatheter heart valve (THV) positioning at the aortic annulus, under-expansion, valve under-sizing, presence of native leaflet overhang, paravalvular aortic regurgitation following THV deployment, and the presence of bicuspid valves.^7,8^

In randomized TAVR trials, THV embolization occurs at a rate of 0.5–1%.^9^ A retrospective, international, multi-centre trial registry, known as TranscatheteR HeArt Valve EmboLization and Migration (TRAVEL) study, reported an incidence rate of 0.92%, of which 217 cases (79.5%) migrated towards the ascending aorta, while 56 (20.5%) did so to the LV.^10^

Currently, the new-generation TAVR devices have shown favourable results in PNAVR. In a meta-analysis of 13 studies with severe inoperable PNAVR patients treated with TAVR, a self-expandable valve was used in 79% of the patients, and the remaining 21% of the cases were resolved with a balloon-expandable valve.^4^ The success rate of these devices ranged from 77 to 100%, with conversion to SAVR reported in only six (2.5%) cases. Additionally, a 7% incidence rate of second valve implantation was observed due to either device migration or severe post-procedural aortic regurgitation.^4^ The self-expandable properties of these devices were considered to provide stability during device placement and ensure anchoring of the prosthesis, even in the absence of significant calcification.

Although the literature mentions that self-expandable valves are safer in PNAVR due to the anatomical characteristics (105 mm annulus perimeter), we opted for a balloon-expandable valve because the annulus was outside the anatomical range allowed for an expandable valve. Also, in balloon-expandable valves, it is possible to over-expand and reach a safe oversizing for the implant.

Retrograde valve migration generally requires surgery to retrieve the heart valve and for conversion to SAVR. Nevertheless, a practical strategy that prevents the need for SAVR is to immediately deliver a second valve to the correct aortic annular position before proceeding to surgical removal of the migrated valve.^11,12^

In our patient, despite performing a 15% oversizing, the anatomical characteristics influenced valve migration. However, the immediate migration and autonomous repositioning of the valve in the aortic annulus made it possible to successfully place a second valve with greater oversizing. According to the literature, this is the first case of valve migration to the LV that was resolved with valve-in-valve therapy, without the need to switch to a surgical approach for SAVR.

The autonomous migration of the valve in reverse position carries the risk of obstructive shock, which, in this patient, led to cardiac arrest, as valves failed to open during systole. In this situation, immediate introduction of the guidewire into the LV should be considered crucial as it offers the possibility of performing a valve-in-valve procedure.

## Conclusion

Although SAVR is a Class I indication for PNAVR, TAVR is a feasible treatment strategy in selected high-risk patients with PNAVR but is associated with a considerable risk of valvular migration. Surgery is the standard approach in valve migration. However, there are some exceptional scenarios like ours, where an interventional procedure may help the patient to avoid a major operation and also help to stabilize the patient quickly.

## Supplementary Material

ytad554_Supplementary_Data

## Data Availability

The data underlying this article are available in the article and in its online Supplementary material.

## Lead author biography

Dr Juan F. Garcia-Garcia graduated in interventional cardiology at Centro Médico Nacional 20 de Noviembre. He currently serves as a professor of the interventional cardiology course at CMN 20 de Noviembre and is the professor of the cardiology service course at the General Hospital of Mexico. Dr Garcia-Garcia possesses extensive experience in complex coronary interventions and transcatheter aortic valve replacement.
